# Maternal Fish Consumption, Mercury Levels, and Risk of Preterm Delivery

**DOI:** 10.1289/ehp.9329

**Published:** 2006-09-25

**Authors:** Fei Xue, Claudia Holzman, Mohammad Hossein Rahbar, Kay Trosko, Lawrence Fischer

**Affiliations:** 1 Harvard School of Public Health, Harvard University, Boston, Massachusetts, USA; 2 Department of Epidemiology and; 3 Integrative Toxicology, Michigan State University, East Lansing, Michigan, USA

**Keywords:** fish consumption, pregnancy, preterm delivery, mercury

## Abstract

**Background:**

Pregnant women receive mixed messages about fish consumption in pregnancy because unsaturated fatty acids and protein in fish are thought to be beneficial, but contaminants such as methylmercury may pose a hazard.

**Methods:**

In the Pregnancy Outcomes and Community Health (POUCH) study, women were enrolled in the 15th to 27th week of pregnancy from 52 prenatal clinics in five Michigan communities. At enrollment, information was gathered on amount and category of fish consumed during the current pregnancy, and a hair sample was obtained. A segment of hair closest to the scalp, approximating exposure during pregnancy, was assessed for total mercury levels (70–90% methylmercury) in 1,024 POUCH cohort women.

**Results:**

Mercury levels ranged from 0.01 to 2.50 μg/g (mean = 0.29 μg/g; median = 0.23 μg/g). Total fish consumption and consumption of canned fish, bought fish, and sport-caught fish were positively associated with mercury levels in hair. The greatest fish source for mercury exposure appeared to be canned fish. Compared with women delivering at term, women who delivered before 35 weeks’ gestation were more likely to have hair mercury levels at or above the 90th percentile (≥ 0.55 μg/g), even after adjusting for maternal characteristics and fish consumption (adjusted odds ratio = 3.0; 95% confidence interval, 1.3–6.7).

**Conclusion:**

This is the first large, community-based study to examine risk of very preterm birth in relation to mercury levels among women with low to moderate exposure. Additional studies are needed to see whether these findings will be replicated in other settings.

High levels of maternal fish consumption during pregnancy have been associated with longer gestation (Borod et al. 1999; Olsen and Secher 1990, 2002; Olsen et al. 1989, 1991, 1992, 2000; Reddy et al. 1994), increased birth weight (Borod et al. 1999; Grandjean et al. 2001; Olsen and Secher 1990, 2002; Olsen et al. 1992, 1993; Reddy et al. 1994), reduced risk of intrauterine growth retardation (IUGR) (Olsen and Secher 2002; Olsen et al. 2000), and lower prevalence of pregnancy-induced hypertension (Olsen and Secher 1990). These reported beneficial effects from fish consumption have been attributed to large amounts of omega-3 fatty acids in fish. But fish and seafood, especially species high in the food chain, are also potential sources of exposure to pollutants such as methylmercury that may adversely affect pregnancy outcomes. Thus, advising pregnant women on fish consumption requires consideration of potential risks as well as benefits (Cohen et al. 2005).

Methylmercury bioaccumulates in predatory fish (Gilbert and Grant-Webster 1995); when these fish are eaten, approximately 95% of the methylmercury is absorbed via the gastrointestinal tract (Aberg et al. 1969; Miettinen et al. 1971). There is concern that levels of methylmercury that are not toxic to adults may pose a hazard to the developing fetus. Total mercury in hair, which is approximately 70–90% methylmercury (Berglund et al. 2005; Chen et al. 2002), has been evaluated in relation to newborn neurologic abnormalities, primarily in studies of heavily exposed populations (Murata et al. 2004; Myers et al. 2003). However, a recent study of mother–infant dyads in a population with relatively lower levels of mercury exposure reported a small negative effect of increasing *in utero* mercury exposure on a visual recognition memory test at 6 months of age (Oken et al. 2005).

Studies on birth weight and total mercury levels, measured in maternal hair or cord blood, have produced mixed results, with some reporting an inverse relationship (Foldspang and Hansen 1990; Sikorski et al. 1986) and others showing no association (Grandjean et al. 2001; Lucas et al. 2004). In addition, one study of maternal occupational exposure to metallic mercury found no effect on birth weight (Fu 1993). Lower birth weights can result from poorer fetal growth and/or delivery at an earlier gestational age. Of the four studies that have examined levels of mercury in relation to gestational age at birth, three measured total mercury in cord blood (Foldspang and Hansen 1990; Grandjean et al. 2001; Lucas et al. 2004), and one focused on women exposed to metallic mercury through work (Fu 1993); all reported no association. Because preterm delivery rates are on the rise in the United States (Goldenberg et al. 1998), and there is some evidence that fish oils may help reduce the risk of preterm delivery (Oakeshott et al. 2002; Olsen and Secher 2002; Olsen et al. 2000), it is particularly important to examine risks associated with pollutants in fish.

Overall, there is limited information from U.S. populations regarding maternal mercury levels and fish consumption and their effects on pregnancy outcome such as preterm delivery. In the Pregnancy Outcomes and Community Health (POUCH) Study (Holzman et al. 2001), a prospective study of biologic and psychosocial factors related to preterm delivery (PTD), the authors had an opportunity to examine maternal mercury levels in hair at mid-pregnancy in > 1,000 women recruited from five communities in Michigan, a state that borders four of the five Great Lakes. Fish consumption and corresponding mercury exposure were evaluated in relation to the risk of PTD.

## Materials and Methods

### Population

**Figure 1:**
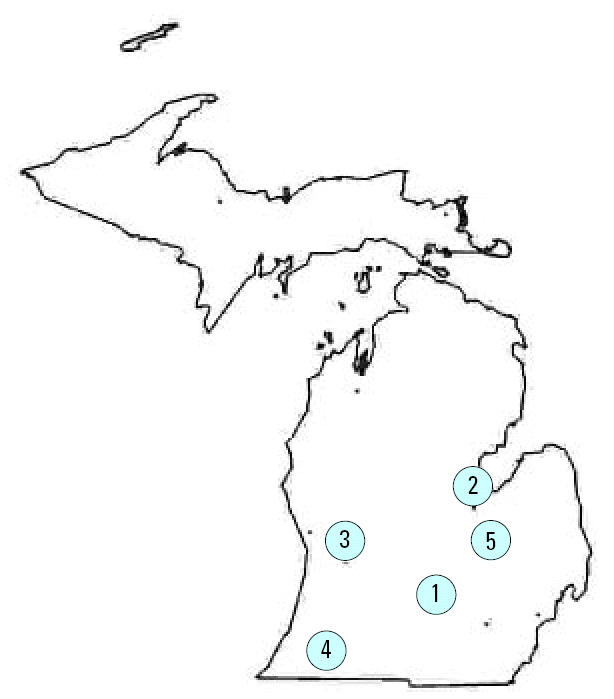
Location of the five Michigan communities in this study.

The POUCH Study recruited women from 52 participating prenatal clinics located in five Michigan communities, each of which include urban, suburban, and rural areas (Holzman et al. 2001) (Figure 1). Women were enrolled in the 15th to 27th week of pregnancy, with approximately 70% before the 24th week. Eligibility criteria included screening for maternal serum alpha-fetoprotein (MSAFP) levels between 15 and 22 weeks of pregnancy, > 14 years of age, competency in English, singleton pregnancy with no known congenital or chromosomal anomalies at the time of recruitment, and no prepregnancy diabetes mellitus. A total of 1,226 women were enrolled in the POUCH cohort during the first part of the study (8 September 1998 through 31 July 2001). All were included in these analyses, except for five because of loss to follow-up and an additional 197 because hair samples were not available (69 declined hair sampling and 129 had hair that was < 7.6 cm long or in a woven hairstyle). Mid-pregnancy hair mercury levels were assessed in the remaining 1,024 cohort women. The study protocol was approved by human subjects review boards at participating institutions. Before enrollment, all participants provided written consent.

Due to Health Insurance Portability and Accountability Act (HIPAA) regulations, it was not possible to determine an exact response rate or compare characteristics of participants with those of women who declined enrollment in the POUCH study. However, we were able to compare POUCH study data with data recorded on birth certificates of women who delivered in the five study communities in 2000. Ethnic-specific analyses (white non-Hispanic, African American), weighted by the proportion of women enrolled from each community, showed that the POUCH sample was very similar to community mothers on most factors measured—age, parity, education levels, and the proportions of women with Medicaid insurance, preterm delivery, previous stillbirth, previous preterm infant, and previous low birth weight infant. The one exception was the percentage of African Americans > 30 years of age, which was lower in POUCH (14%) than in community birth certificates (21%).

### Gestational age

Gestational age at delivery was determined by date of first day of the last menstrual period (LMP), or a gestational age estimate from an early ultrasound (≤ 25 weeks), the latter used when the two estimates disagreed by > 2 weeks. Early ultrasound data were available for 93% of women.

### Maternal characteristics and fish consumption

Information on maternal characteristics including age, ethnicity, education, Medicaid insurance status, and smoking was collected through in-person interviews and self-administered questionnaires at enrollment. As part of the interview, women were asked “During this pregnancy how often have you eaten any of the following fish: shellfish, canned fish, other fish you purchased at a store or restaurant [referred to as bought fish], sport-caught fish in Michigan waters, and some other fish?” For each fish category, respondents were asked about their number of meals per day, week, month, or previous 6 months. Six months was used as an option for infrequent consumers of fish and was thought to capture the period from conception to study interview for most women in the study. The data were then scaled so that all fish consumption—fish categories and total—could be expressed as meals per 6 months, thereby describing levels of fish intake in approximately the first 6 months of pregnancy. Total fish consumption was calculated by summing consumption of all categories of fish and shellfish.

### Mercury levels in hair

At enrollment, approximately ≥ 100 strands of hair were cut close to the scalp from the posterior vertex region of women with hair at least 7.6 cm in length. A segment of hair closest to the scalp, approximating exposure during pregnancy, was assessed for total mercury levels. The length of segment used varied by gestational week at enrollment, assuming average hair growth of approximately 1.3 cm per month (Saitoh et al. 1967). Before analysis, hair was washed with acetone and water to remove mercury deposited from external sources. Cold vapor atomic absorption spectrometry (CVAAS; model M6000; Cetac Technology, Omaha, NE) was used to quantify total mercury levels in hair. It is estimated that around 70–90% of mercury in hair is methylmercury (Chen et al. 2002). Because of this and other factors, researchers consider total mercury levels in hair to be a useful biomarker of exposure to methylmercury (Berglund et al. 2005; Chen et al. 2002).

### Analytic strategy

We used generalized linear models (GLM) to assess the relationships between mercury levels in hair, maternal characteristics, and fish consumption. We used multicovariate logistic regression to evaluate the association between maternal mercury levels and risk of preterm delivery (< 37 weeks’ gestation), moderate preterm delivery (35–36 weeks’ gestation), and very preterm delivery (< 35 weeks’ gestation). Threshold levels for mercury effects were tested at quintile cutoffs and at the 90th percentile. To meet the underlying assumptions of the GLM, mercury levels were transformed to natural log scale (micrograms per gram) for analyses and then transformed back to mercury levels (micrograms per gram) for display in tables. All analyses were conducted using SAS 9.0 software (SAS Institute Inc., Cary, NC). On examining self-reports of total fish consumption, we found three women who had consumed > 300 fish meals in the 6 months corresponding to the first half of pregnancy. These outliers could not be verified and were removed from the analyses.

## Results

At the time of enrollment, 41% of women were < 25 years of age, 42% had ≤ 12 years of education, and 43% were insured by Medicaid, a health-related public assistance program (Table 1). The percentage of women from racial/ethnic backgrounds other than non-Hispanic white and African American was only 9%. The “other” ethnic groups were included with non-Hispanic whites in the final models. Alternative analyses excluding these other groups showed similar results to that of the more inclusive final models.

In approximately the first 6 months of pregnancy, 11% of women in this sample did not eat any fish, 25% ate two fish meals or fewer, and only 50% ate more than nine fish meals. The mean level of total fish consumption was 19.6 meals/6 months, considerably higher than the median (50th percentile = 9.0 meals/6 months), suggesting a right skewing of the distribution (Table 2). Canned fish was the most frequently consumed fish category, with 25% of women eating ≥ 12 meals/6 months, followed by bought fish, with 25% eating ≥ 6 meals/6 months. Only 9.2% of women reported consumption of sport-caught fish during the first 6 months of pregnancy.

Mercury levels in maternal hair ranged from 0.01 to 2.50 μg/g, with a mean of 0.29 μg/g and a median of 0.23 μg/g. Approximately 20% of women had levels > 0.38 μg/g (Table 3). Mercury levels were divided into quintiles and total fish consumption and consumption of each fish category were divided into four levels: 0 meals/6 months (reference group), 1–5 meals/6 months, 6–23 meals/6 months, and ≥ 24 meals/6 months. Women with higher levels of total fish consumption were more likely to have mercury levels in the upper quintiles (Table 3). The mean and median hair mercury levels for the 109 women who did not consume fish during this period in pregnancy were 0.15 μg/g and 0.13 μg/g, respectively. Interestingly, 10% of women who reported not eating fish during pregnancy had hair mercury levels in the 4th and 5th quintiles.

**Figure 2:**
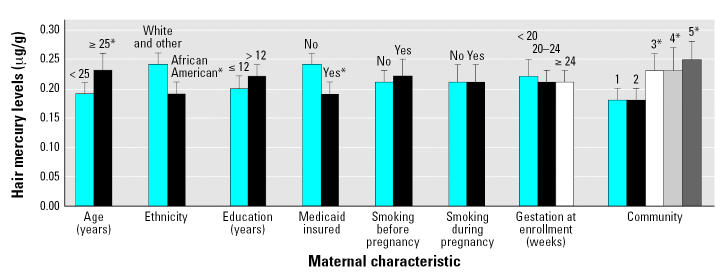
Mercury levels (95% CI) in hair collected at mid-pregnancy according to maternal characteristics, adjusted for all the other covariates in the figure and total fish consumption. Mercury levels were transformed to a natural log and then transformed back as exp [mean of ln(mercury)].< br>*p < 0.05.

In multicovariate analyses, higher mercury levels were significantly associated with older maternal age (≥ 25 years), white and “other” ethnicity, not being insured by Medicaid, and residing in communities 3, 4, and 5 (Figure 2), even after adjusting for total fish consumption. Maternal mercury levels were not significantly related to gestational week at enrollment or smoking before or during pregnancy. We reevaluated the association between total fish consumption and mercury levels in a model that included maternal covariates related to mercury levels in hair (Table 4). The adjusted mean mercury continued to be significantly higher in women who consumed fish compared with that in women who did not consume fish in the first 6 months of pregnancy, and mean mercury levels increased as levels of fish consumption increased. In another model that included consumption of each fish category along with the other maternal covariates related to mercury levels in this sample, consumption of canned fish, bought fish, and sport-caught fish were each positively associated with mercury levels in maternal hair. Adjustment for gestational week at enrollment did not appreciably alter these associations.

In the final analyses, we assessed maternal mercury levels at mid-pregnancy in relation to gestational week at delivery. Women who delivered very preterm (< 35 weeks) were more likely to have had hair mercury levels at or above the 90th percentile (0.55–2.5 μg/g) than were women who delivered at term (≥ 37 weeks), even after adjusting for maternal characteristics and total fish consumption [odds ratio (OR) = 3.0; 95% confidence interval (CI), 1.3–6.7] (Table 5). These results remained relatively unchanged in models that adjusted for gestational week at enrollment and fish categories. The association between maternal mercury levels and very preterm delivery was not evident at lower threshold levels of mercury (i.e., quintile cut-points), and mercury levels were not associated with delivery of a moderately preterm infant (35–36 weeks).

## Discussion

Women enrolled in the POUCH Study resided in communities surrounded by the Great Lakes, but their levels of fish consumption in the first 6 months of pregnancy would be considered moderate to low relative to populations that subsist on fish. Despite the modest levels of fish consumption, there was strong evidence that mercury levels in maternal hair were higher when fish consumption levels were higher.

A positive correlation between fish consumption and mercury levels in pregnant women has been reported in studies conducted in European countries (Bjornberg et al. 2003; Daniels et al. 2004; Oskarsson et al. 1994) and the Amazon basin of South America (Bruhn et al. 1995; Hacon et al. 2000). Fewer data are available from pregnant populations in North America. Two studies from Canada in areas close to the Great Lakes showed a positive association between fish consumption and mercury levels in maternal hair (Morrissette et al. 2004; Muckle et al. 2001). Other studies from the Great Lakes area have observed a positive relationship between fish consumption and mercury levels in nonpregnant women of child-bearing age (Nadon et al. 2002), anglers and fish eaters (Cole et al. 2004), Native Americans (Gerstenberger et al. 1997), Montreal sportfishers of Asian origin (Kosatsky et al. 1999b), and the general population (Kosatsky et al. 2000; Mahaffey and Mergler 1998). The concordance of the information provided from these and other studies indicates that in diverse regions of the globe, consumption of fish is a major source of methylmercury exposure in humans.

In one study, methylmercury was the most commonly identified pollutant in sport-caught fish (Anderson et al. 2004). Canned tuna has been implicated as one of the main foods contributing to total mercury intake (Legrand et al. 2005; U.S. Department of Health and Human Services and U.S. Environmental Protection Agency 2006). Because of variation in methylmercury levels by fish type and location, studies in different settings have tried, as we tried in this study, to link mercury levels to consumption of particular types of fish. In anglers and Native Americans residing in the Great Lakes region, levels of mercury have been shown to be positively associated with consumption of sport-caught fish (Cole et al. 2004; Gerstenberger et al. 1997; Kosatsky et al. 1999a; Nadon et al. 2002). A study by the Wisconsin Division of Public Health reported that women of child-bearing age who ate sport-caught fish had higher mercury levels in hair than those of women who did not eat sport-caught fish, but the difference was not statistically significant (Knobeloch et al. 2005). Morressette et al. (2004) reported that bought fish, including canned fish, were important sources of mercury exposure during pregnancy. These studies are consistent with our findings of higher hair mercury levels in pregnant women who consumed greater amounts of canned fish, sport-caught fish, and bought fish.

As anticipated, hair mercury levels in POUCH Study mothers were lower than levels found in heavily exposed, fish-eating communities. Across studies the unit of measure used to report hair mercury levels varies—parts per million, milligrams per kilogram, or micrograms per gram—but these units are equivalent and can be directly compared. The median hair mercury level in the Faroe Islands sample was 4.5 ppm, and in the Seychelles 5.9 ppm, almost double the highest mercury level observed in the POUCH Study (Davidson et al. 1999; Grandjean et al. 1992). In a study of Swedish women of child-bearing age, 127 women reported consuming, on average, four fish meals/week and had a median hair mercury level of 0.70 mg/kg (Bjornberg et al. 2005). A study of 150 pregnant women from varied locations in Alaska found somewhat lower levels: The median hair mercury was 0.47 mg/kg and the mean was 0.72 mg/kg (Arnold et al. 2005). Two studies—one from Massachusetts, the other from southwest Québec—reported mercury levels more similar to those in our findings. The first study included 135 pregnant women recruited from an HMO in eastern Massachusetts (Oken et al. 2005). On average, women in that study consumed slightly more fish (1.2 meals/week) than did women in the POUCH Study (0.82 meals/week), and had slightly higher hair mercury levels (mean = 0.55 ppm; 10% exceeded 1.2 ppm) than did those in the POUCH Study (mean = 0.29 μg/g; 10 % exceeded 0.54 μg/g). In the Québec study of 159 pregnant women, mercury was assessed in sequential centimeters of hair to represent levels during different months of pregnancy (Morrissette et al. 2004). Among women who consumed two or more fish meals/month, mean hair mercury levels were about 0.20 μg/g at the 5th–6th month of pregnancy. Differences in maternal mercury levels across these studies may be explained by differences in both amount and types of fish consumed.

In the POUCH Study, maternal characteristics of older age, ethnicity of white and other, not being insured by Medicaid, and residing in three of the five communities were significantly associated with higher maternal mercury levels after adjusting for fish consumption. The reasons for these associations are unclear and may include other lifestyle factors, mercury exposures not related to fish, and residual confounding from incomplete adjustment for fish types, portion size, and method of preparation. The observed higher mean mercury level among women without Medicaid insurance—a marker of higher socioeconomic status—is consistent with a recently released report by the Wisconsin Division of Public Health, which also found higher mercury levels in association with higher socioeconomic status (i.e., college education and annual household income > $75,000) (Knobeloch et al. 2005). It is unlikely that the community differences in mercury levels within our study reflect variations in pollutant levels in local fish, because most of the fish consumed are not from local waters. About 10% of women in the POUCH Study reported not consuming fish during pregnancy but had hair mercury levels in the top two quintiles. This suggests that their diet histories may have been inaccurate or there may be sources of exposure, other than fish, that are uncommon but deserve consideration.

Our study is the first to report an association between delivery at < 35 weeks’ gestation and maternal hair mercury levels ≥ 0.55 μg/g (upper 10th percentile). The biologic mechanism supporting this finding requires further investigation. It has been shown that methylmercury produces oxidative stress at the cellular level (Sanfeliu et al. 2003), which may be a contributing factor. In addition, methylmercury can influence shape, aggregation, and levels of platelets (Hornberger and Patscheke 1989; Macfarlane 1981) and thromboxane (Caprino et al. 1983). These effects may potentiate underlying pathology related to maternal vascular diseases in pregnancy. Within the POUCH Study we plan to examine maternal biomarkers of endothelial dysfunction and will explore whether they are related to maternal mercury levels.

Several methodologic differences could account for the inconsistency between our study results and those of previous studies that did not detect an association between mercury levels and gestational age at delivery (Foldspang and Hansen 1990; Fu 1993; Grandjean et al. 2001; Lucas et al. 2004). Our study correlating fish consumption and maternal mercury levels in hair is the largest in the United States to date, providing greater statistical power to detect moderate associations (OR = 3.0) with an outcome that is less frequent, such as delivery at < 35 weeks. Studies using blood levels of mercury (maternal, cord) reflect variations in recent exposure (Foldspang and Hansen 1990; Grandjean et al. 2001; Lucas et al. 2004), whereas mercury in hair is relatively stable and mid-pregnancy hair levels better represent average exposure across the first half of pregnancy. The POUCH Study women had considerably lower mercury levels relative to those of women in other studies, allowing for the testing of lower thresholds. Only one study assessed preterm delivery as an outcome (Fu 1993), and the other three considered mean gestational age at delivery which may have obscured mercury effects in the tail of the distribution (i.e., very preterm) (Foldspang and Hansen 1990; Grandjean et al. 2001; Lucas et al. 2004). In addition, other studies did not adjust for fish consumption, a potential confounder in populations with high levels of fish consumption. Researchers have hypothesized that omega-3 fatty acids in fish can prolong gestation by down-regulating the synthesis of prostaglandin (PG) E2 and PGF2a, and promoting the synthesis of PGI2 and PGI3, thus leading to a more relaxed myometrium (Ferretti et al. 1988; Hansen and Olsen 1988).

Major strengths of the present study include the large number of pregnant women participating, the prospective design, and the use of hair as an index of methylmercury exposure. Hair levels of total mercury represent a longer window of exposure than those of blood levels. This study also had several limitations: First, information on fish consumption was based on recall, which can lead to inaccurate estimates. However, the recall period was focused on eating habits during pregnancy, a time when women are often more aware of their dietary patterns. Because the reports of fish consumption were obtained well before the outcome of the pregnancy, there is little concern about differential recall bias. Second, maternal interviews did not include information on portion size of fish meals and methods of preparation. Factoring in these details might further strengthen correlations between levels of fish consumption and levels of mercury in hair. Third, women were not asked about number of dental amalgam fillings. Total mercury measured in maternal hair may include 10–30% inorganic mercury, the major form of mercury in amalgam fillings (Spencer 2000). However, it is widely accepted that in populations similar to those we have studied, food—particularly fish—is the major exposure source of methylmercury, the primary component of total mercury found in hair. In addition, several studies have reported little or no relationship between hair total mercury and exposure to amalgams (Berglund et. al. 2005; Bjornberg et al. 2003; Hansen et al. 2004; Pesch et al. 2002; Tulinius 1995).

An important limitation is our lack of information on maternal levels of other pollutants commonly found in fish (e.g., organo-chlorines). Women with higher mercury levels in hair may have been exposed to higher levels of other contaminants, such as polychlorinated biphenyls (PCBs) and dichlorodiphenyl-dichloroethylene (DDE), a metabolite of DDT. Hair mercury levels and blood PCB and DDE levels have all been found to be elevated in high-level fish consumers compared with people who eat less fish (Kosatsky et al. 1999a, 1999b). Based on the estimate of average daily dietary exposure to contaminants for approximately 120,000 adults from the Nurses’ Health Study and the Health Professional Follow-up Study, intraindividual exposures to mercury and DDE were found to be moderately correlated among women (*r* = 0.27) and men (*r* = 0.17) (MacIntosh et al. 1996). We tried to adjust for other pollutants in fish and for the potentially beneficial aspects of fish (e.g., omega-3 fatty acids) by including total fish consumption in our final analyses. After adjustment, the positive association between maternal mercury levels in hair and risk of very preterm delivery remained, but we cannot rule out residual confounding by other pollutants. Previous studies have failed to find a link between preterm delivery risk and levels of PCBs (Berkowitz et al. 1996; Longnecker et al. 2005) or DDE (Farhang et al. 2005; Fenster et al. 2006; Torres-Arreola et al. 2003). The one exception is a report of excess preterm delivery among women with high blood levels of DDE (Longnecker et al. 2001). This study used stored blood samples from the Collaborative Perinatal Project, a U.S. cohort assembled in 1959 through 1966. The DDE levels associated with preterm birth were considerably higher than DDE levels found in U.S. women of reproductive age today.

Our study reinforced previous findings suggesting that fish consumption is a major source of mercury exposure for pregnant women. Although much attention has been focused on pollutants in locally caught fish, and fish advisories are not uncommon, we found that only a small percentage of pregnant women, < 10%, consumed sport-caught fish during pregnancy. The greatest fish source for mercury exposure appeared to be canned fish, both because it was consumed more and, per meal, it was among the fish categories associated with the highest levels of mercury in maternal hair. The observed relationship between elevated mercury levels and increased risk of very preterm delivery is a new finding and requires caution in interpretation. Although the study sample was large, the number of women who delivered before 35 weeks of pregnancy was small (*n* = 44), and more studies are needed to test this association.

## Figures and Tables

**Table 1:** Maternal characteristics in the 1,024 POUCH Study women with mercury levels measured in hair at mid-pregnancy.

Maternal characteristics	No. (%)
Age (years)	
< 25	422 (41)
≥ 25	602 (59)
Ethnicity	
White	752 (73)
African American	183 (18)
Other	89 (9)
Education (years)^a^	
≤ 12	430 (42)
> 12	590 (58)
Medicaid insured^a^	
No	587 (57)
Yes	436 (43)
Smoking before pregnancy	
No	751 (74)
Yes	268 (26)
Smoking during colspan="2" pregnancy
No	859 (84)
Yes	161 (16)
Weeks of pregnancy at enrollment	
< 20	141 (14)
20–23	606 (59)
≥ 24	277 (27)
Community	
1	393 (38)
2	198 (19)
3	148 (15)
4	100 (10)
5	185 (18)

a Data missing for 1–5 women.

**Table 2:** Number of fish meals consumed in approximately the first 6 months of pregnancy.

Maternal fish consumption	Mean ± SD	Range	25th percentile	50th percentile	75th percentile
Total fish	19.6 ± 28.2	0–214.5	2.0	9.0	26.0
Shellfish^a^	3.7 ± 10.1	0–182.5	0.0	1.0	3.0
Canned fish^a^	8.5 ± 16.5	0–182.5	0.0	2.0	12.0
Bought fish^a^	6.3 ± 18.5	0–182.5	0.0	1.0	6.0
Sport-caught fish^a^	0.7 ± 4.9	0–90.0	0.0	0.0	0.0
Other fish^a^	0.4 ± 6.0	0–182.5	0.0	0.0	0.0

a Data missing for 5–8 women.

**Table 3:** Distribution (%) of maternal hair mercury levels by levels of total fish consumption in approximately the first 6 months of pregnancy.

	Quintiles of mercury levels (μg/g) in hair based on the entire study sample				
Total fish consumption [no. of meals/6 months (*n*)]	1st (0.01–0.12)	2nd (0.13–0.19)	3rd (0.20–0.26)	4th (0.27–0.38)	5th (0.39–2.50)
0 (109)	48.6	29.4	11.9	7.3	2.8
1–5 (267)	19.5	30.3	23.6	15.4	11.2
6–23 (347)	13.8	15.0	22.5	23.9	24.8
≥ 24 (288)	11.1	16.3	16.0	27.4	29.2

**Table 4:** Unadjusted and adjusted mean mercury levels in maternal hair in relation to levels of fish consumption (total fish and fish categories).

	Mean mercury [μg/g (95% CI)]^a^	
Levels of fish consumption [no. of meals/6 months (*n*)]	Unadjusted	Adjusted
Total fish^b^		
0 (109) (referent)	0.13 (0.11–0.14)	0.11 (0.10–0.13)
1–5 (267)	0.20 (0.18–0.21)*	0.17 (0.16–0.18)*
6–23 (347)	0.25 (0.23–0.27)*	0.21 (0.20–0.23)*
≥ 24 (288)	0.28 (0.26–0.30)*	0.25 (0.23–0.27)*
Shellfish^c^		
0 (435) (referent)	0.18 (0.17–0.20)	0.28 (0.21–0.36)
1–5 (372)	0.25 (0.23–0.27)*	0.34 (0.26–0.44)
6–23 (174)	0.29 (0.26–0.32)*	0.34 (0.27–0.44)
≥ 24 (37)	0.25 (0.20–0.32)*	0.33 (0.24–0.46)
Canned fish^c^		
0 (307) (referent)	0.16 (0.15–0.18)	0.24 (0.19–0.32)
1–5 (306)	0.23 (0.21–0.24)*	0.31 (0.24–0.41)*
6–23 (258)	0.28 (0.26–0.31)*	0.37 (0.29–0.48)*
≥ 24 (146)	0.28 (0.26–0.32)*	0.38 (0.29–0.49)*
Bought fish^c^		
0 (472) (referent)	0.20 (0.18–0.21)	0.29 (0.22–0.38)
1–5 (262)	0.24 (0.22–0.26)*	0.33 (0.26–0.44)*
6–23 (196)	0.27 (0.24–0.30)*	0.35 (0.26–0.45)*
≥ 24 (90)	0.26 (0.22–0.30)*	0.32 (0.24–0.41)
Sport-caught fish^c^		
0 (920) (referent)	0.22 (0.21–0.23)	0.21 (0.20–0.23)
1–5 (69)	0.27 (0.23–0.32)*	0.24 (0.20–0.28)
6–23 (19)	0.34 (0.25–0.47)*	0.28 (0.20–0.38)
≥ 24 (14)	0.40 (0.28–0.58)*	0.35 (0.25–0.50)*
Other fish^b^		
0 (996) (referent)	0.22 (0.21–0.23)	0.22 (0.20–0.23)
1–5 (17)	0.34 (0.24–0.48)	0.30 (0.22–0.41)
6–23 (4)	0.21 (0.11–0.43)	0.22 (0.12–0.42)
≥ 24 (6)	0.40 (0.23–0.70)*	0.34 (0.20–0.57)

a Mercury levels were transformed to a natural log and then transformed back as exp [mean of ln(mercury)].

b Total fish consumption is adjusted for maternal age, ethnicity, Medicaid status, and community. Models of fish categories are adjusted for these same maternal factors and for other fish categories.

c Data on consumption of each category of fish were missing for 1–7 women.

* *p* < 0.05 for test of difference in means with 0 as referent group.

**Table 5:** The association between high mercury levels in maternal hair sampled at mid-pregnancy [≥ 90th percentile (0.55–2.50 μg/g)] and risk of preterm delivery.

	OR (95% CI)	
Pregnancy outcome (*n*)	Unadjusted	Adjusted^a^
Term [≥ 37 weeks (923)]		
All preterm [< 37 weeks (101)]	1.1 (0.6–2.2)	1.55 (0.7–2.9)
Moderately preterm [35–36 weeks (57)]	0.3 (0.1–1.4)	0.4 (0.1–1.9)
Very preterm [< 35 weeks (44)]	2.4 (1.1–5.1)	3.0 (1.3–6.7)

a Adjusted for total fish consumption, maternal age, ethnicity, Medicaid status, and community.
